# Gold Nanoclusters Display Low Immunogenic Effect in Microglia Cells

**DOI:** 10.3390/nano11051066

**Published:** 2021-04-21

**Authors:** Joanna Sobska, Magdalena Waszkielewicz, Anna Podleśny-Drabiniok, Joanna Olesiak-Banska, Wojciech Krężel, Katarzyna Matczyszyn

**Affiliations:** 1 Advanced Materials Engineering and Modelling Group, Wroclaw University of Science and Technology, WybrzezeWyspianskiego 27, 50-370 Wroclaw, Poland; joanna.sobska@pwr.edu.pl (J.S.); joanna.olesiak-banska@pwr.edu.pl (J.O.-B.); 2 Institut de Génétique et de Biologie Moléculaire et Cellulaire, Department of Development and Stem Cells, 1 Rue Laurent Fries, 67404 Illkirch, France; podlesny@igbmc.fr; 3 Institut de la Santé et de la Recherche Médicale, U 1258, 67404 Illkirch, France; 4 Centre National de la Recherche Scientifique, UMR 7104, 67404 Illkirch, France; 5 Université de Strasbourg, 67404 Illkirch, France; 6 Polish Center for Technology Development—Port Lukasiewicz, Stabłowicka 147, 54-066 Wrocław, Poland; magdalena.waszkielewicz@port.lukasiewicz.gov.pl

**Keywords:** nanoparticles, cytotoxicity, immunological response, biodistribution

## Abstract

Gold nanoparticles hold a great promise for both clinical and preclinical applications. The major factors impeding such applications are toxicity of new nanomaterials including e.g., pro-apoptotic activities or inflammatory effects, but also their potential to accumulate in the body or inadequate absorption, distribution, metabolism and excretion (ADME) profiles. Since such adverse effects depend on the size, form and coating of nanomaterials, the search for new, less toxic nanomaterials with low tendency to accumulate is highly active domain of research. Here, we describe optical and biological properties of Au18 gold nanoclusters (NCs), small gold nanoparticles composed of 18 atoms of gold and stabilized with glutathione ligands. These nanoclusters may be suitable for in vivo applications owing to their low toxicity and biodistribution profile. Specifically, using lactate dehydrogenase (LDH) test in P19 cell line we found that Au18 NCs display low toxicity in vitro. Importantly, using primary microglial cells we showed that at low concentrations Au18 NCs display anti-inflammatory signaling on evidence of reduced interleukin 1-β (IL1-β) levels and unchanged levels of tumor necrosis factor (TNF-α) or Ym1/2. Such effect was dose dependent as higher concentrations of Au18 NCs induced expression of pro-inflammatory cytokines and suppression of anti-inflammatory cytokine Ym1/2, pointing, thus, to global inflammatory activity. Finally, we also showed that within 3 days Au18 NCs can be completely eliminated from the liver reported as the major target organ for accumulation of gold nanoparticles. These data point to a potential of gold nanoparticles for further biomedical studies.

## 1. Introduction

Nanoclusters are a class of metallic nanoparticles (NPs) with particularly small size (1–2 nm) and distinct quantum-size effects, which allow a discrete electronic structure. Their spectra exhibit molecular-like one-electron transitions rather than collective excitations present in metallic gold nanocrystals. The special electronic and geometric structures of nanoclusters give rise to many other noteworthy properties such as chirality, magnetism, redox potential and photovoltaic properties [1,2,3].

The gold nanoclusters (NCs) gain increasing interest in the field of biological applications [4,5]. However, there is still not enough knowledge about neither the toxicity of metal nanoclusters nor their endogenous biological activities, which could compromise future medical applications. Despite a generally good biocompatibility of gold, the use of bare, non-coated gold nanoparticles is associated with a formation of aggregates and subsequent accumulation in the liver or other organs [6], which in turn is the causal factor of a liver necrosis [7,8]. Similarly, the toxicity of adverse biological activities was reported for different types of chemically synthesized Au nanoparticles of different sizes usually bigger than 10 nm [9,10,11]. In contrast, Au NCs were shown to display none or only low toxicity [12,13,14]. These and other studies pointed to critical importance of the size, shape and coating of nanoparticles for their toxicity with a strong tendency for a reduction of toxic effects with a decrease of particles size [15,16]. However, in contrast to the low toxicity of small gold nanoparticles, their higher potency to elicit an immunological reaction was reported, one of the potential hurdles in biomedical applications of nanomaterials. Accordingly, small nanoparticles could more easily penetrate macrophage cells and provoke stronger inflammatory responses [9]. However, such data contrasted with other reports on chemically produced Au NCs (containing specific surface modifiers or stabilizers), which showed just the contrary. Thus, Shukla et al. or Guével et al. reported that Au NCs did not increase pro-inflammatory cytokines levels [17] and did not induce lymphocyte proliferation nor activation of natural killer cells via dendritic cells, despite being actively taken-up by the latter ones [14]. In addition to the size of Au NPs, the inflammatory potential was also shown to be determined by the shape and the type of chemical surface modifiers of nanoparticles [18]. Importantly, a few studies also pointed to the possibility that Au NCs may prevent or decrease inflammation including neuroinflammation. Although the exact mechanism of such activity is not known, it was suggested for citrate-stabilized, small, but not large nanoparticles, that they may induce anti-inflammatory effects by direct interactions with interleukin 1-β (IL-1β) attenuating thereby its activity [19].

Another important determinant of the utility of nanoparticles for biomedical applications is their absorption, distribution, metabolism and excretion (ADME) profile, which should be optimized to allow NPs to display a broad or a highly restricted distribution, and in particular to assure their clearance from the organism, once they have accomplished their function. It has been demonstrated that gold nanoparticles and nanoclusters display different ADME profiles and activities depending on their size, shape and coating. However, a general trend which emerged from such studies pointed to the reduced long-term accumulation and better excretion profiles for small, 2–10 nm gold spherical nanoparticles or nanoclusters [20]. An obstacle in the interpretation of the size-function relationship in these studies results from relatively wide range of nanoparticle or nanocluster sizes used for such analysis.

Thus, small nanoparticles or gold nanocluster, which are biologically inert or display limited or beneficial (e.g., anti-inflammatory or antiviral) biological activities, should be particularly useful for the development of new tools for future nanomedicine. Here, we report the protocol for generation of small sized nanoclusters with narrow range of sizes, which display low cell toxicity, no inflammatory activity at low concentrations and, finally, do not accumulate in the brain and liver but are effectively removed from the body by renal pathway.

## 2. Materials and Methods

### 2.1. Instrumentation

The absorption was measured in 10 mm quartz cuvette, with the two-beam spectrophotometer JASCO V-670 (Tokyo, Japan) and fluorescence measurements were recorded with spectrofluorometer Hitachi F-4500 (Tokyo, Japan) (with excitation and emission slits set to 2 and 5 nm resolution, respectively). High-resolution transmission electron microscopy (TEM) was conducted with FEI Tecnai G2 20 X-TWIN (Tokyo, Japan), with acceleration voltage 200 kV.

### 2.2. Preparation of Au_18_(SG)_14_ Nanoclusters

The Au_18_(SG)_14_ nanoclusters (called hereafter as Au18 NCs) were synthesized following Ghosh protocol [21]. Briefly, to 1.2 mL 0.368 mM methanol solution of gold (III) chloride trihydrate (HAuCl_4_•3H_2_O, 99.999%, Sigma Aldrich, Poznan, Poland), 1.8 mL water and 300 mg of glutathione (GSH, purity ≥ 98.0%, Sigma Aldrich, Poznan, Poland) were added. The solution was sonicated to dissolve glutathione (the color changed from yellow to almost colorless). It was diluted with 96 mL of methanol (Avantor Performance Materials, Sigma Aldrich, Poznan, Poland) The solution was then stirred for 10 min. Then, 4.5 mL of 220 mM aqueous solution of sodium cyanoborohydride (NaBH_3_CN, 95%, Sigma Aldrich, Poznan, Poland) was added to it. After 30 min of vigorous stirring, the precipitate was collected and washed 3 times with methanol through centrifugal precipitation to remove the remaining precursors. The precipitate was dissolved in water and centrifuged to remove unreacted thiolate. Finally, the solution was evaporative dried to obtain a pale red powder, re-dissolved in Milli-Q water and left for several weeks in darkness (aging process).

Additionally, a mixture of various sizes of AuSG nanoclusters was prepared following the protocol by Negishi et al. [22]. Briefly, 1.0 mmol of GSH was added into 50 mL of methanolic solution of HAuCl_4_ (0.005 M). Then, the aqueous solution of NaBH_4_ (0.2 M, 12.5 mL) was poured rapidly into this mixture under vigorous stirring. The mixture was allowed to react for an hour. The reaction mixture was collected and centrifuged to obtain a precipitate. The product was washed repeatedly with methanol. Finally, Au:SG clusters were dried to obtain dark-brown powder.

### 2.3. Polyacrylamide Gel Electrophoresis (PAGE)

The PAGE electrophoresis was performed in discontinuous gel, as described in [23]. The gel was produced from acrylamide monomers with the final concentrations of 18.3%T; 4.2%C and 7.5%T; 2.6%C for separating and stacking gel, respectively (%T is total monomer concentration and %C is concentration of the cross-linking). The nanoclusters were dissolved in a 5% (*v/v*) glycerol/water solution. The electrophoresis was carried out for 13 h at a constant voltage mode (150 V).

### 2.4. Lactate Dehydrogenase (LDH)Cytotoxicity Assay

P19 cells were seeded on 96-well plate with density 10,000 cells/well in the Dulbecco’s Modified Eagle Medium (DMEM) medium (DMEM (1 g/L glucose) + 5% FCS 3396 + 5% FCS delipidated + Gentamycinum 10 μg/mL). They were treated with Au18 NCs after 24 h and then they were incubated for next 3, 24 and 48 h (37 °C, 5% CO_2_). In addition, the cells were treated with 1% (*v/v*) water as a control of the solvent used to prepare the nanocluster solution. The Pierce LDH cytotoxicity assay kit was purchased from Thermo Scientific ™ (ref. 88954) and used to determine cell death. The standard protocol assays were performed according to the manufacturer’s instructions. Briefly, 50 µL of medium was transferred to the new 96-well plate. Then 50 µL of reaction mixture was added and incubated 30 min at room temperature. Finally, the reaction was stopped adding 50 µL of stop solution. Absorbance was measured at 490 nm and 680 nm using a microplate reader.

### 2.5. Primary Microglial Cells

Primary culture of microglia was prepared from newborn mice at postnatal day 3 (P3). Whole brains devoid of meninges and blood vessels, were dissociated by mild mechanical trituration in cold Phosphate-buffered saline (PBS). The isolated cells were cultured for 12 days in DMEM with GlutaMAX (Gibco, Grand Island, NY, USA) supplemented with 10% Fetal Calf Serum (FCS, Hyclone Co., Logan, UT, USA) and 1 × PenStrept. Then, the mixed glial cultures were shaken on an orbital shaker at 150 rpm for 4 h to remove microglial cells. Cells were cultured overnight on precoated poly-L-lysine (Sigma, Lezennes, France) dish. After 24 h microglia were treated with Au18 NCs at 6.0 × 10^−6^ mg/mL, 6.0 × 10^−4^ mg/mL, 6.0 × 10^−3^ mg/mL corresponding respectively to 0.006 ppm, 0.6 ppm, 6.0 ppm (ppm—μg of particles per milliliter) or lypopolysaccharyde (LPS; 125 ng/mL) and H_2_O for 3 h.

### 2.6. RNA Isolation and Quantitative RT-PCR

After treatments with NCs, LPS or PBS, cells were harvested in lysis buffer provided by RNeasy Micro Kit (Qiagen, Duesseldorf, Germany). RNA was isolated following the manufacturer’s instruction. RNA quality was assessed by Nano Drop (Thermo Fischer Scientific, Illkirch-Graffenstaden, France). For qRT-PCR, 400 ng of RNA was transcribed into cDNA using oligo-dT primers and Transcriptor Reverse Transcriptase Kit (Roche, Meylan, France). cDNA was transferred into 96-well Multiply PCR plate (Roche, France) with appropriate volume of SybrGreen master mix (Qiagen, Duesseldorf, Germany) and reaction was performed in Light Cycler 480 (Roche, Meylan, France). Following gene-specific primers were used at 5 pM concentration (Table 1). The amount of transcript was evaluated relatively to the amount of transcript of the housekeeping gene—acidic ribosomal phosphoprotein P0 (Rplp0 or 36B4). Statistical analysis was performed using relative expression values against 36B4, using Student t-test (significant for *p*-value < 0.05).

### 2.7. Biodistribution

For the in vivo experiments we used C57BL6N mice (Charles River, Écully, France). Mice were housed in 12 h light/dark cycle with unlimited access to water and food. Au18 NCs were injected intraperitoneal (IP) at final concentration of 10 mg/kg and mice were sacrificed at 3, 24 and 72 h after treatment (*n* = 3 mice for each time point). Vehicle treatment was performed in control group of mice (*n* = 3), which were sacrificed 3 h after treatment. Liver and kidney samples were weighted and lyophilized. For further analyses samples were chemically dissolved in aqua regia at 90 °C. Concentration of Au18 NCs was measured using inductively coupled plasma optical emission spectrometer (ICP-OES; model 720, Agilent, Santa Clara, CA, USA).The experiments were approved by local ethics committee (authorisation No. 2018030111543287) and accredited by the French Ministry for Superior Education and Research in accordance with the Directive of the European Union Council (2010/63/EU), and were carried in compliance with the guidelines of CNRS and the French Agricultural and Forestry Ministry (decree 87848).

### 2.8. Statistical Methods

GraphPad software was used to perform graphs and statistical analysis. The statistics for the cytotoxicity experiment and anti-inflammatory effects of Au18 NCs were performed using one-way ANOVA, whereas biodistribution statistics was determined using two-way ANOVA with organ type (liver, kidney and brain) and time (0, 3 and 24 h) as independent measures and quantity of gold as dependent variable. Post-hoc analyses were performed using Fisher’s least significant difference (LSD) test. Student t-test was used to compare effect of LPS and Au18 NCs at 6 ppm.

## 3. Results and Discussion

### 3.1. Characterization of Au18 NCs

**Figure 1:**
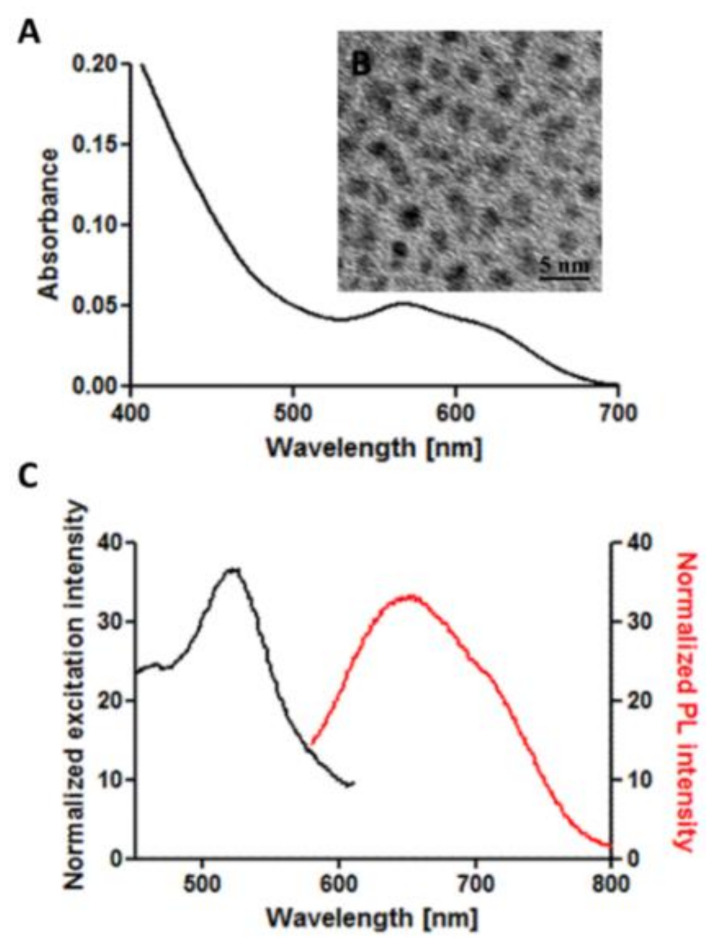
(**A**) Absorption spectrum of an aqueous solution of Au18. (**B**) TEM image of Au18 nanoclusters (NCs) solution. (**C**) Excitation (black) and emission (red) spectra of Au18 NCs. The sample was excited at 540 nm and the maximum of the emission was at 640 nm.

The aqueous solution of nanoclusters was obtained and comprehensively characterized with spectroscopic methods. NCs present absorption bands at 560 nm and 625 nm, which correspond with the spectra of Au18SG14 presented by Jin [24] (Figure 1A). The size of the nanoclusters was determined by TEM, and the average diameter was defined as 2.16 ± 0.21 nm (Figure 1B). In order to confirm the homogeneous size of our nanoclusters we ran PAGE electrophoresis experiment (Figure S1). A mixture of Au NCs stabilized with glutathione [22] was applied as a reference sample. Figure S1 as well as TEM images and absorption measurements show that Au18 sample presents a narrow size distribution and size of nanoclusters corresponding to nanoclusters of ~18 gold atoms. Such precise control over the size of nanoparticles is not possible with larger metal nanoparticles, as well as most of the other inorganic nanoparticles applied as diagnostic agents in medicine. The emission spectrum (Figure 1B) presents a broad photoluminescence band with the maximum at 640 nm (excitation 540 nm). Fluorescence in a red-wavelength range is favorable from the point of view of imaging applications in vivo, as was recently shown for glutathione-stabilized nanoclusters [25]. Moreover, nanoclusters present broad range of fluorescence excitation (450–600 nm), thus can be easily combined with other fluorophores for multiplexed imaging (e.g., with green-emitting probes excited in a blue wavelength range). The fluorescence quantum yield (QY) of the nanoclusters, calculated basing on the comparison with Oxazine 170 fluorescence [26], was around 4%. Importantly, NCs without any further post-synthetic purification, when stored in a powder form preserve their optical properties for several months.

### 3.2. Cytotoxicity

The use of nanoparticles in biology requires to determine the safe dose in use, first in cell cultures and then in living organisms. Cytotoxicity of NCs coated with glutathione and consisting of 25 Au atoms were tested by Zhao et al. with 3-[4,5-dimethylthiazol-2-yl]-2,5-diphenyltetrazolium bromide (MTT) assay and the survival of the Vero, A549 and Madin-Darby canine kidney (MDCK) cells was almost 100% after 24 h incubation [27]. Other studies, such as [28], also showed low toxicity of nanoclusters when covered with GSH for concentrations lower than 100 µMin cultures of normal ATII and cancerous A549 cells.

**Figure 2:**
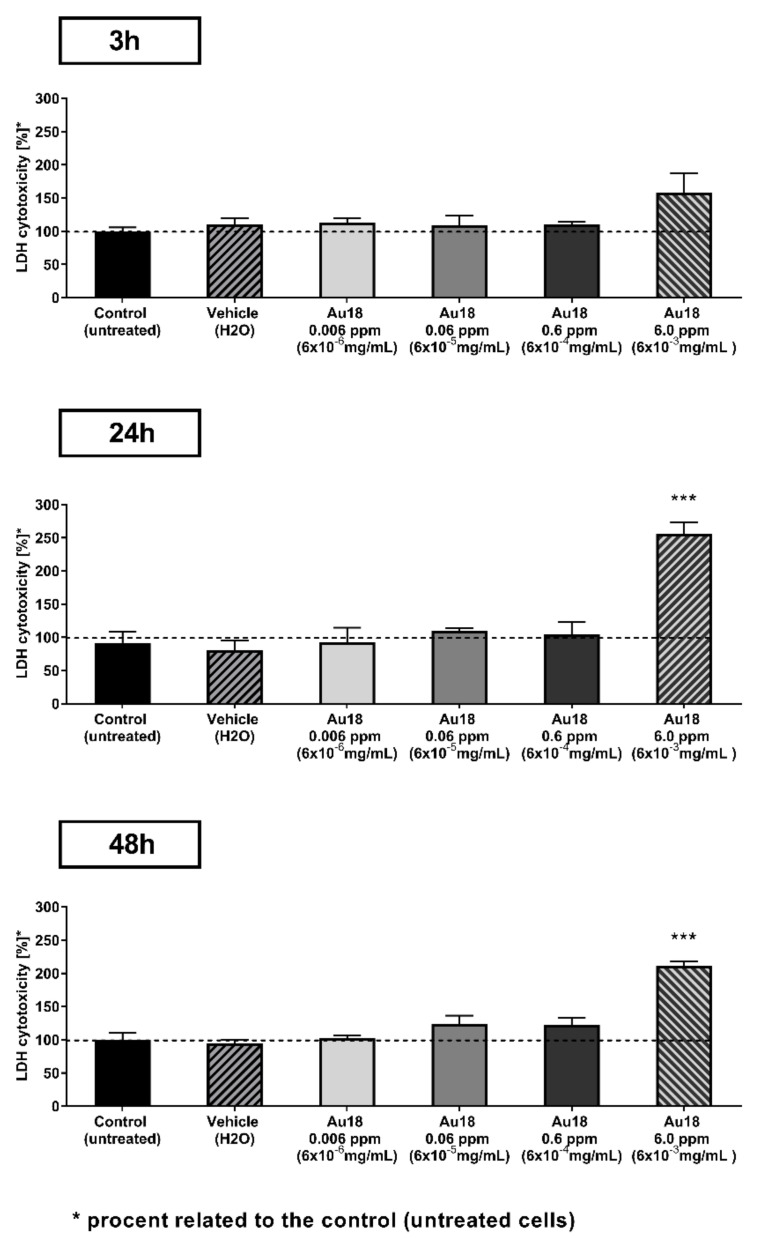
Cytotoxicity of Au18 NCs on P19 cells in lactate dehydrogenase (LDH) test. P19cells were incubated with culture medium containing NCs at concentrations between 0.006 ppm to 6.0 ppm and were compared to vehicle (H_2_O) treated cells (negative control) at 3, 24 and 48 h. Non-treated cells were also reported as no effect of vehicle was observed. Each experimental group consisted of *n* = 3 independent samples and error bars represent standard error of means. *** *p* < 0.001 as compare to vehicle treated cells.

We performed the cytotoxicity tests with Au18 NCs and P19 cells using LDH assay, which is based on measuring the amount of LDH. Cell death causes the cell membrane to become permeable to the enzyme, which consequently get into the cell medium. Thus, it is less prone to produce unreliable results for glutathione-capped nanoclusters, where glutathione may take part in redox reactions (e.g., such as reduction of MTT in MTT assays). After 3 h incubation of NCs at concentrations in the range between 0.006 ppm and 0.6 ppm we observed good survival of cells at level comparable to control sample of vehicle (H_2_O) treated cells as revealed by absence of significant effect of treatment in one-way ANOVAanalyses (F(4,10) = 1.92, ns; Figure 2 top panel). However, such statistical analysis for longer periods of incubation revealed significant effect of treatment at 24 h (F(4,10) = 18.77, *p* < 0.001; Figure 2 middle panel) and 48 h (F(4,10) = 32.1, *p* < 0.001; Figure 2 bottom panel). Post-hoc analyses using Fisher’s LSD test pointed that such difference reflects a significant increase of Au18 NCs at the highest 6.0 ppm concentration when compared to vehicle treated cells.

### 3.3. Inflammatory Response

**Figure 3:**
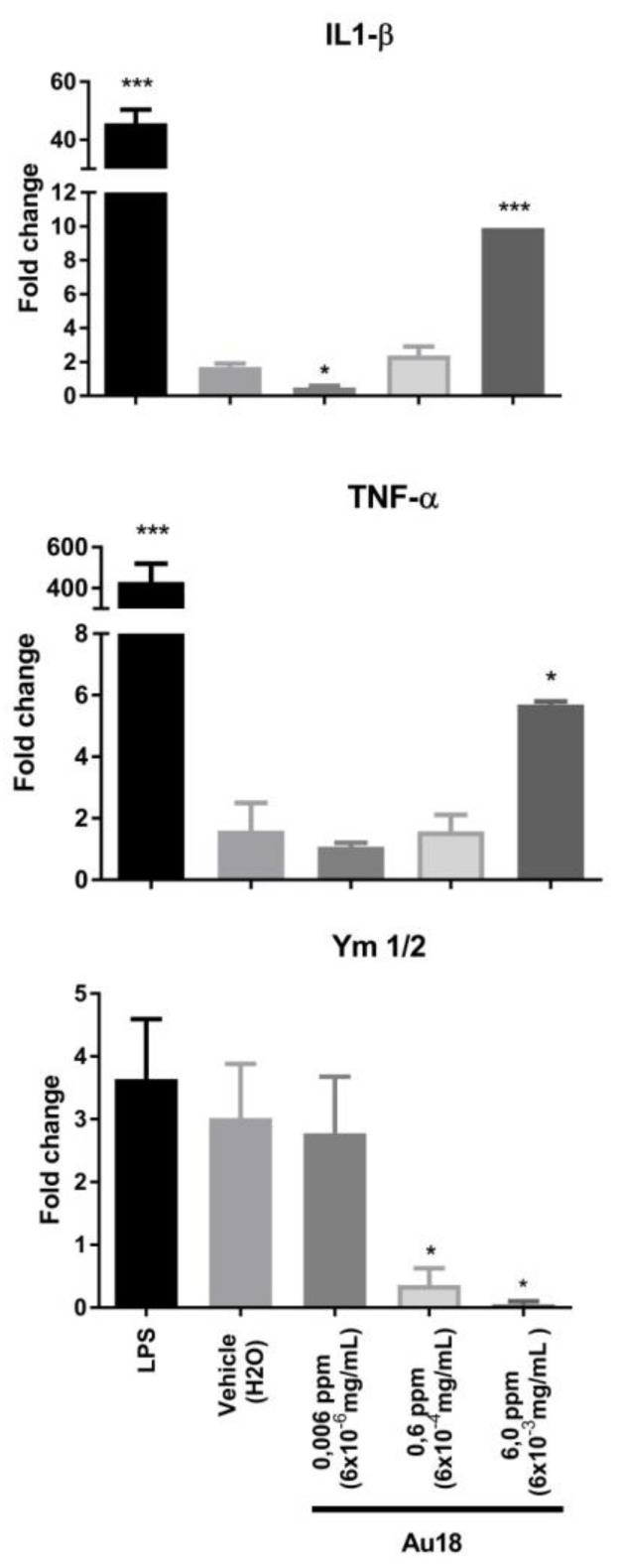
Inflammatory responses of mouse primary microglia cells in response to Au18 NCs. Primary microglia cells were treated with Au18 NCs (0.006 ppm, 0.6 ppm, 6.0 ppm) or lypopolysaccharyde (LPS) (125 ng/mL) and cultured during 3 h. The expression of interleukin 1-β (IL-1β), tumor necrosis factor (TNF-α) and Ym1/2 was examined using RT-qPCR (*n* = 3/group, * *p* < 0.05; *** *p* < 0.001 as compare to untreated cells).

Nanoparticles were frequently reported to elicit inflammatory responses, which might compromise their utility in clinical setting. Since such effects are determined by multiple factors including size, shape, surface charge and chemical composition it is not easy to predict activity of new nanomaterials, which remains to be verified experimentally [29]. The inflammatory response has been studied with many types of gold nanoparticles. However, there are only few papers address specifically gold nanoclusters (nanoparticles with diameter smaller than 2 nm) [30]. To address this point, we investigated the effect of the Au18 NCs on the expression of cytokines in primary cultures of mouse microglial cells as a model of diverse myeloid phagocytic cells which all share a number of common functional and molecular features [31]. Statistical analyses using one-way ANOVA revealed significant effect of treatment on expression of each of the studied cytokines: pro-inflammatory IL1-β (F(3,12) = 77.54, *p* < 0.001), and tumor necrosis factor (TNF-α; F(3,9) = 12.79, *p* < 0.01) as well as anti-inflammatory cytokine Ym1/2 (F(3,12) = 4.7, *p* < 0.05). Post-hoc analysis revealed that IL1-β displayed bimodal response (Figure 3, top panel), with almost 70% suppression of its expression in response to low quantities 0.006 ppm (6.0 × 10^−6^ mg/mL) of Au18 NCs and progressive increase of IL1-β expression with increasing amounts of Au18 NCs. Thus, significant 10-fold increase of IL1-β was observed at 6.0 ppm of Au18 NCs. Expression of TNF-α remained unaffected at low concentrations of Au NCs, but significantly increased only in the presence of 6.0 ppm, the highest tested concentration of nanoclusters (Figure 3, middle panel). Consistent with the suppression of inflammatory signaling by the low concentration of Au18 NCs, there was no significant decrease of anti-inflammatory cytokine Ym1/2 in the same treatment conditions (Figure 3, bottom panel). Expression of Ym1/2 was, however completely suppressed at 6.0 ppm, the highest concentration of Au18 NCs at which a strong induction of pro-inflammatory cytokines IL1-β and TNF-α was observed. Such data indicate that effects of Au18 NCs on inflammatory responses is dose-dependent with at low dose of 0.006 ppm Au18NCs display anti-inflammatory activities associated with suppressed IL1-β whereas at high concentrations of 6.0 ppm they display pro-inflammatory effects observed. Importantly, even if the highest doses elicited marked expression of pro-inflammatory cytokines IL1-β and TNF-α, such responses remained at least 10 times weaker than inflammatory signaling induced by 125 ng/mL of LPS, our positive control of activation of microglial cells (*p* < 0.001 for Student t-test comparison of LPS and 6 ppm Au18 NCs effects for IL1-β and TNF-α; Figure 3). Interestingly, LPS did not affect expression of anti-inflammatory Ym1/2, whereas high doses of Au18 NCs significantly suppressed its expression suggesting different mechanisms through which high doses of Au NCs and LPS modulate inflammatory signaling.

Literature data [9,11] present the absence of pro-inflammatory activities of low doses of Au NCs, similarly to the ones reported previously in cell culture models in vitro [17,19] and rodents in vivo [19,32]. In addition, suppression of IL1-β expression by low doses of Au18 NCs supports anti-inflammatory activity of Au NCs reported previously, although through potentially different mechanism. Thus, whereas Sumbayev et al. suggested that effects of Au NCs on IL1-β are mediated by direct interaction of nanoparticles with this cytokine [19], Jeon et al. showed that Au NCs may directly interact with IkappaB kinase, inhibiting thereby global nuclear factor-kappa B (NFkB) signaling and expression of its downstream targets including both, IL1-β and TNF-α [33]. In our study we cannot exclude such interactions, but a decrease of IL1-β but not TNF-α transcript, indicates possibility that Au18NCs influence specifically transcriptional control of IL1-β and, not globally, the NFkB signaling pathway, and that such a control involves transcriptional events. Our data on anti-inflammatory effects of Au18 NCs in microglia, the resident brain macrophages, are of a direct relevance for research into neuroinflammation, one of the major factors contributing to the pathogenesis of several neurodegenerative or psychiatric disorders [34]. Importantly, whereas anti-inflammatory effects induced by 20 nm Au NPs were recently reported to prevent cognitive deficits in the streptozotocin model of sporadic model of Alzheimer diseases [32], Au NCs were not investigated.

Finally, our data indicate that higher doses of Au NCs efficiently induce pro-inflammatory effects similar to those reported for naked [11] or small Au NPs [9] and several other types of gold nanoparticle (reviewed recently by Dykman and Khlebtsov [35]). The activation of immunological response and associated inflammation would be of course deleterious in neurodegenerative diseases, but could be beneficial for generation of vaccines. Thus, the use of high doses or immunogenic gold nanoparticles was proposed as basis for new vaccine adjuvants [35]. In consequence, the latter application would necessitate fine control of biodistribution of nanoparticles to prevent their accumulation which, in turn, could induce or accelerate ageing-dependent neuroinflammation and neurodegeneration.

Present observations reconcile reports of low to high toxicity of AuNPs reported by Yen et al. [9], who used only high concentrations (10 ppm) of Au NPs, and the absence of such activities reported by other teams. In addition to size, concentrations, route of administration etc., an important factor which may determine NP activities is their coating. Thus, considering that glutathione was reported as anti-inflammatory agent [36] we cannot exclude that anti-inflammatory or the low-grade inflammatory activity of Au18 NCs results partially from glutathione coating. However, it remains little probable that glutathione is the key determinant of such activity as doses reported to act as anti-inflammatory were as high as 10 mM, which is 129 times more than 76,8 µM of glutathione concentration introduced as a coating of present Au18 NCs. In line with this possibility is also observation that anti-inflammatory activity ofAu18 NCs was limited to IL1-β whereas Limongi et al. [36] reported wider range of cytokines affected including IL1-β and TNF-α.

### 3.4. Biodistribution

One of the major concerns in the use of nanoparticles for clinical applications, is their optimal ADME profile and in particular possibility of their elimination from the organism after they accomplished specific task(s). Studies conducted by different groups reported strong dependence of nanoparticle ADME profile on the size, shape and chemical surface characteristics. For example, large nanoparticles (with a diameter between 4 nm and 200 nm) were shown to accumulate mainly in the liver and spleen [7,37,38], whereas small nanoparticles and nanoclusters displayed more ubiquitous distribution with possibility of crossing blood brain barrier, but also with frequent cases of liver accumulation and toxicity [37,38,39].

**Figure 4:**
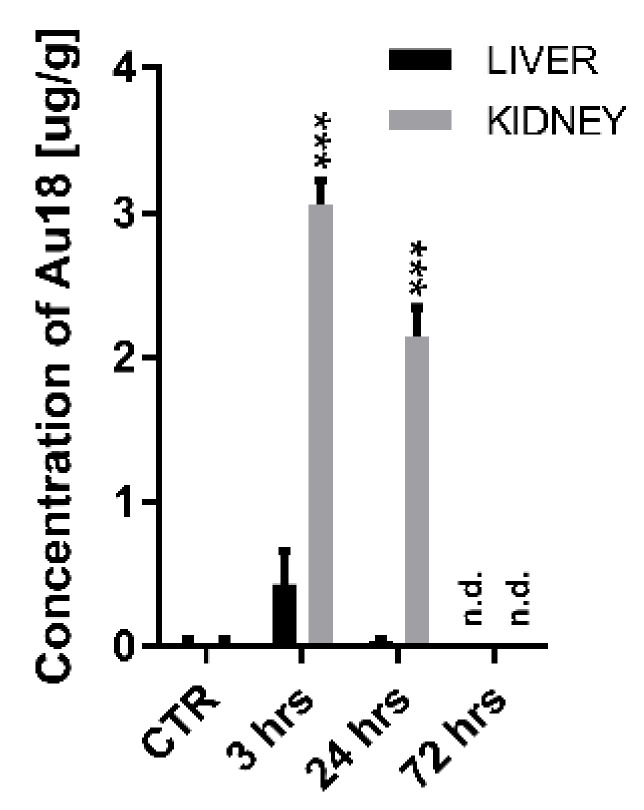
Biodistribution of Au18 NCs in mice. Au concentration measured with inductively coupled plasma optical emission spectrometer (ICP-OES) in non-treated control mice (CTR) and at 3, 24 and 72 h post intraperitoneal injections. Values are represented as mean ± SEM (*n* = 3/time/organ, *** *p* < 0.001 as compared to negative control, CTR).

Here we investigated biodistribution and retention of Au18 NCs focusing on liver as the most consistently reported target organ for nanoparticle accumulation, kidney as the key organ in NPs elimination process and brain. As indicated by strong time x organ interaction in two-way ANOVA analyses performed for liver, kidney and brain in control non-treated mice and at 3 and 24 h (F(4,18) = 41.28, *p* < 0.001)the biodistribution of AuNCs displayed different dynamics depending on the type of studied organ. Post-hoc analyses indicated that at 3 h after IP injection, Au18 NCs were detected in kidney attaining ~3.0 μg/g and liver (0.4 µg/g; *p* = 0.051), whereas at 24 h Au18 were detected only in kidney (2.2 μg/g) (Figure 4). Importantly, at 72 h after injection, Au18 NCs were below detection level in both organs indicating latent, but efficient elimination of Au18 NCs from the organism possibly through renal pathway. In support of this possibility high levels (16.4 μg/µL) of Au18 NCs were detected in the urine at 24 h after administration, but not detected after 72 h. Importantly, Au18 NCs were not detected in the brain at 3 and 24 h after injection of Au18 NCs (the 72 h time-point was not evaluated). Such observation is significant as it indicates that in the physiological conditions Au18 NCs can circulate in the organism over limited period of time without penetrating blood-brain barrier. Instead, in a number of infections such as cytokine storm associated with COVID-19 infection [40] or during systemic inflammation associate with neurodegeneration [41] blood-brain barrier is opened and most probably permeable to Au18 NCs. In such pathological conditions, anti-inflammatory or low pro-inflammatory profile of Au18 NCs, which we observed in microglial cells, can be considered as beneficial or at least not harmful.

Present data support rapid liver and kidney redistribution of Au18 NCs after their intraperitoneal application, but also provide evidence for their efficient elimination from these organs within 72 h. Such observations are in agreement with efficient elimination of small-size spherical nanoparticles from the organism [20].

## 4. Conclusions

Here we show that gold nanoclusters hold a promise for biomedical applications due to their low toxicity, good elimination from the liver and kidney, but also the absence of pro-inflammatory effects. Importantly, such features are strongly dependent on the concentration of Au18 nanoparticles as illustrated by anti-inflammatory effects at low doses and pro-inflammatory effects of high concentrations of Au18 nanoclusters. We cannot exclude, however, that some of such anti-inflammatory activity may reflect presence of glutathione coating on Au18 NCs. Moreover, combination of low toxicity in neural cells and concentration-controlled inflammatory response with good optical properties of nanoclusters (red-wavelength range fluorescence and high photostability) favors their potential application in bioimaging.

## Figures and Tables

**Table 1:** Combinations of forward and reverse primers used in real-time PCR expression analysis.

Gene		
36B4	Forward sequence (5´→3´) Reverse sequence (5´→3´)	ACCCTGAAGTGCTCGACATC AGGAAGGCCTTGACCTTTTC
TNF-α	Forward sequence (5´→3´) Reverse sequence (5´→3´)	CTTCTGTCTACTGAACTTCGGG CAGGCTTGTCACTCGAATTTTG
Il1-β	Forward sequence (5´→3´) Reverse sequence (5´→3´)	ACGGACCCCAAAAGATGAAG TTCTCCACAGCCACAATGAG
Ym1/2	Forward sequence (5´→3´) Reverse sequence (5´→3´)	CAGGGTAATGAGTGGGTTGG CACGGCACCTCCTAAATTGT

## Data Availability

Data is contained within the article or Supplementary Material.

## Supplementary Materials

The following are available online at https://www.mdpi.com/article/10.3390/nano11051066/s1, Figure S1. (A) PAGE electrophoresis of AuSG nanoclusters of various sizes, synthesized with a protocol [22] and AuSG synthesized with a protocol [21], identified as Au18. (B) Absorption spectrum of Au18 NCs, (C) absorption spectrum of Au18NCsfrom publication [24]. Comparison of PAGE with [22] and comparison of the spectrum (B) with [24] and references therein, allows to assign nanocluster sizes in fraction P1 as a mixture of Au10-Au18 and the second sample as Au_18_SG_14_.
