# Supplementary material for: Gold Nanoclusters Display Low Immunogenic Effect in Microglia Cells
Source: Nanomaterials (Basel). 2021 Apr 21;11(5):1066. doi: 10.3390/nano11051066 (PMC8143360; doi:10.3390/nano11051066)
Supplement: Supplementary file 1 [file nanomaterials-11-01066-s001.zip › nanomaterials-1183905-supplementary.pdf]

nanomaterials

MDPI

Article

Gold Nanoclusters Display Low Immunogenic Effect in Microglia Cells

Joanna Sobska [1,2,3,4,5], Magdalena Waszkielewicz [6], Anna Podleśny-Drabiniok [2,3,4,5], Joanna Olesiak-Banska [1], Wojciech Krężel [2,3,4,5,*] and Katarzyna Matczyszyn [1,*]

1 Advanced Materials Engineering and Modelling Group, Wroclaw University of Science and Technology, WybrzezeWyspianskiego 27, 50-370 Wroclaw, Poland; joanna.sobska@pwr.edu.pl (J.S.); joanna.olesiak-banska@pwr.edu.pl (J.O.-B.)
2 Institut de Génétique et de Biologie Moléculaire et Cellulaire, Department of Development and Stem Cells, 1 Rue Laurent Fries, 67404 Illkirch, France; podlesny@igbmc.fr
3 Institut de la Santé et de la Recherche Médicale, U 1258, 67404 Illkirch, France
4 Centre National de la Recherche Scientifique, UMR 7104, 67404 Illkirch, France
5 Université de Strasbourg, 67404 Illkirch, France
6 Polish Center for Technology Development - Port Lukasiewicz, Stabłowicka 147, 54-066 Wrocław, Poland; magdalena.waszkielewicz@port.lukasiewicz.gov.pl
* Correspondence: krezel@igbmc.fr (W.K.); katarzyna.matczyszyn@pwr.edu.pl (K.M.)

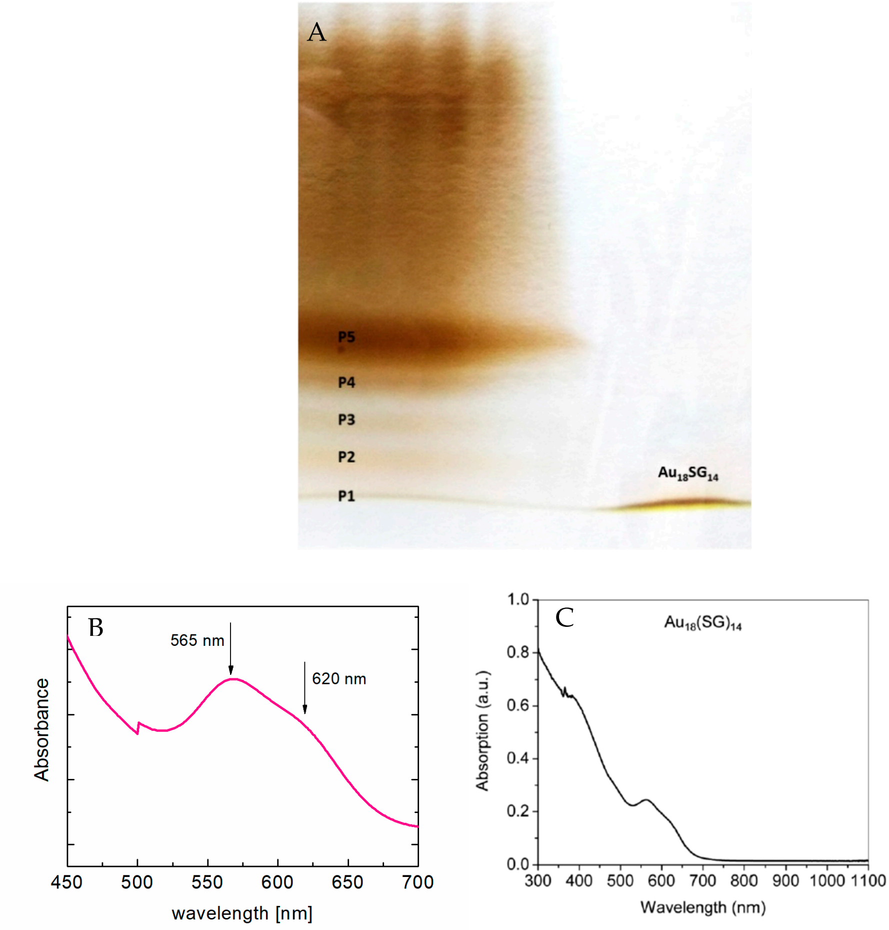

Figure S1. (A) PAGE electrophoresis of AuSG nanoclusters of various sizes, synthesized with a protocol [1] and AuSG synthesized with a protocol [2], identified as $Au_{18}(SG)_{14}$. **(B)** Absorption spectrum of Au18 nanoclusters, **(C)** absorption spectrum of $Au_{18}(SG)_{14}$ from publication [3]. Comparison of PAGE with [1] and comparison of the spectrum B with [3] and references therein, allows to assign nanocluster sizes in fraction P1 as a mixture of Au10-Au18 and the second sample as $Au_{18}SG_{14}$.

References

1. Negishi, Y.; Nobusada, K.; Tsukuda, T.Glutathione-protected gold clusters revisited: Bridging the gap between gold(I)-thiolate complexes and thiolate-protected gold nanocrystals. *J. Am. Chem. Soc.* **2005**, *127*, 5261–5270, doi:10.1021/ja042218h.
2. Ghosh, A.; Udayabhaskararao,T.; Pradeep, T. One-step route to luminescent Au 18SG 14 in the condensed phase and its closed shell molecular ions in the gas phase. *J. Phys. Chem. Lett.* **2012**, *3*, 1997–2002, 2012, doi:10.1021/jz3007436.
3. Jin, R. Atomically precise metal nanoclusters: Stable sizes and optical properties. *Nanoscale* **2015**, *7*, doi:1549–1565, 2015, doi:10.1039/c4nr05794e.
